# Supplementary material for: Structure and density of silicon carbide to 1.5 TPa and implications for extrasolar planets
Source: Nat Commun. 2022 Apr 27;13:2260. doi: 10.1038/s41467-022-29762-y (PMC9046200; doi:10.1038/s41467-022-29762-y)
Supplement: Supplementary file 1 — Supplementary Information [file 41467_2022_29762_MOESM1_ESM.pdf]

Supplementary Materials for

Structure and Density of Silicon Carbide to 1.5 TPa and Implications for Extrasolar Planets

Authors: D. Kim,[1,*] R. F. Smith,[2] I. K. Ocampo,[1] F. Coppari,[2] M.C. Marshall,[3] M.K. Ginnane,[3] J. K. Wicks,[4] S. J, Tracy,[5] M. Millot,[2] A. Lazicki,[2] J.R. Rygg,[3] J. H. Eggert,[2] T. S. Duffy[1]

Affiliations:

[1]Department of Geosciences, Princeton University, Princeton, NJ, USA.

[2]Lawrence Livermore National Laboratory, Livermore, CA, USA.

[3]Laboratory for Laser Energetics, University of Rochester, Rochester, NY, USA.

[4]Department of Earth & Planetary Sciences, Johns Hopkins University, Baltimore, MD, USA.

[5]Earth and Planets Laboratory, Carnegie Institution for Science, Washington, DC, USA.

*Corresponding author. Email: donghoonkim86@gmail.com

Supplementary Table 1. Descriptions of target packages.

Shot	Laser	Ablator (µm)	Sample (µm)	Window (µm)	He_α X-ray source	Pinhole Substrate
89310	Omega-60	C[15]Au[1]C[15]	SiC[17]	C[62]	Cu	W
26662	Omega-EP	C[39]	SiC[12.5]	LiF[101.5]	Cu	Ta
27424	Omega-EP	C[40]	SiC[20]	LiF[100.5]	Cu	W
26666	Omega-EP	C[42]	SiC[21]	LiF[101]	Cu	Ta
27425	Omega-EP	C[43]	SiC[20]	LiF[98.5]	Cu	W
27430	Omega-EP	C[37]	SiC[20]	LiF[101.5]	Cu	W
96727	Omega-60	C[22]Au[1]C[16]	SiC[12]	C[60]	Ge	Pt
29131	Omega-EP	C[14]Au[1]C[16.5]	SiC[12]	C[109]	Cu	Pt
29130	Omega-EP	C[15]Au[1]C[12]	SiC[16]	C[105.5]	Cu	Pt
98948	Omega-60	C[17.5]Au[1]C[23.5]	SiC[13]	C[60]	Ge	Pt
96731	Omega-60	C[21]Au[1]C[21.5]	SiC[10]	C[60]	Ge	Pt
96728	Omega-60	C[18.5]Au[1]C[19.5]	SiC[15]	C[55]	Ge	Pt
98953	Omega-60	C[20.5]Au[1]C[22.5]	SiC[16]	C[62]	Ge	Pt
98955	Omega-60	C[21]Au[1]C[24]	SiC[14]	C[57]	Ge	Pt
96735	Omega-60	C[21]Au[1]C[22.5]	SiC[11]	C[55]	Ge	Pt
98954	Omega-60	C[16.5]Au[1]C[20]	SiC[11.5]	C[60]	Ge	Pt
89314	Omega-60	C[18.5]Au[1]C[19]	SiC[10.5]	C[63]	Ge	Pt
89315	Omega-60	C[15]Au[1]C[19.5]	SiC[10]	C[60]	Ge	Pt
89316	Omega-60	C[16.5]Au[1]C[19.5]	SiC[10]	C[60]	Ge	Pt

Numbers in brackets are target dimensions in micrometers. A 1-µm thick gold layer was used as a heat shield and deposited directly onto the diamond ablator for high-stress shots (>400 GPa). The X-ray energies for Cu and Ge He_α X-ray sources are 8.368 keV and 10.249 keV, respectively[1].

Supplementary Table 2. *d*-spacings, densities, and phase assignments of ramp-compressed SiC

Shot	P_x (GPa)	P_s (GPa)[a]	d_{111} (Å)	d_{002} (Å)	d_{022} (Å)	d_{113} (Å)	a(Å)	Density (g/cm^3)	Phase
89310	80(4)	64(4)	2.405(34)	2.060(30)	1.468(16)	1.243(9)	4.13(2)	3.78(5)	B3
26662	140(5)	136(5)	2.131(22)	1.844(19)	-	-	3.69(3)	5.30(13)	B1
27424	178(7)	173(7)	2.098(24)	1.813(20)	-	-	3.63(3)	5.57(14)	B1
26666	245(10)	239(10)	2.064(26)	1.782(16)	-	-	3.55(2)	5.96(10)	B1
27425	303(21)	296(21)	2.032(25)	1.749(19)	1.233(7)	-	3.49(2)	6.27(11)	B1
27430	371(11)	364(11)	1.988(21)	1.710(15)	1.223(7)	-	3.43(2)	6.66(12)	B1
96727	433(15)[b,c]	424(15)[b,c]	-	1.636(19)	-	-	3.27(4)	7.62(28)	B1
29131	470(16)[b]	462(16)[b]	-	1.668(16)	-	-	3.34(3)	7.15(19)	B1
29130	521(16)[b]	513(16)[b]	-	1.669(11)	-	-	3.34(2)	7.15(13)	B1
98948	558(20)[b]	549(20)[b]	-	1.646(19)	-	-	3.29(4)	7.48(27)	B1
96731	611(20)[b]	602(20)[b]	-	1.627(21)	-	-	3.25(4)	7.76(29)	B1
96728	621(20)[b]	611(20)[b]	-	1.611(22)	-	-	3.22(4)	7.98(30)	B1
98953	764(30)[b]	754(30)[b]	1.863(31)	1.603(17)	-	-	3.21(3)	8.05(22)	B1
98955	858(28)[b]	848(28)[b]	1.850(31)	1.594(18)	-	-	3.19(3)	8.21(23)	B1
96735	875(36)[b]	865(36)[b]	-	1.612(26)	-	-	3.22(5)	7.98(37)	B1
98954	996(33)[b]	985(33)[b]	1.820(29)	1.585(26)	-	-	3.16(4)	8.44(32)	B1
89314	1197(38)[b]	1185(38)[b]	-	1.512(19)	-	-	3.02(4)	9.67(38)	B1
89315	1436(45)[b]	1423(45)[b]	1.724(24)	1.492(22)	-	-	2.98(3)	10.07(30)	B1
89316	1507(64)[b]	1495(64)[b]	1.742(26)	1.502(16)	-	-	3.01(3)	9.77(29)	B1

Numbers in parenthesis are one-standard deviation uncertainty in the last digit(s). P_x is the measured longitudinal stress. [a]The pressure along the isentrope, P_s, is calculated assuming Y and γ_0 are 20 GPa and 1, respectively. [b]Targets with diamond windows have an additional potential +50 GPa uncertainty due to diamond strength uncertainty (see Materials and Methods). [c]Shot 96727 at 433 GPa yielded anomalous results and was excluded from equation of state fitting. Specifically, the observed *d*-spacing was 1.636(19) Å, corresponding to a density of 7.62(28) g/cm^3. This is inconsistent with the trend shown in Fig. 5[2,3]. Notably, the *d*-spacing (~1.73 Å) of the diamond peak recorded in this shot is also relatively low, which would be consistent with a stress above 600 GPa. Thus, the stress in this shot is possibly underestimated for unknown reasons.

Supplementary Table 3. Experimental and theoretical equations of state for B1 SiC.

V_0 (Å^3)	K_0 (GPa)	K_0'	EOS, method	References
		Experiment		
66.3 (fixed)	286(20)	3.83(16)	BM (Isentrope)[a]	This work
66.3 (fixed)	265(26)	4.4(3)	Vinet (Isentrope)[a]	This work
66.3(1)	323(3)	3.1(4)	BM, XRD	Ref.[3]
66.3(14)	324(2)	3.3(5)	Vinet, XRD	Ref.[3]
67.5(4)	235(9)	4	BM, XRD	Ref.[2]
		Computation		
65.9	252.3	4.26	Vinet, DFT (LDA)	Ref.[4]
-	240.0	3.63	BM, ab initio	Ref.[5]
62.6	278.4	2.61	BM, DFT (LDA)	Ref.[6]
62.6	266.6	4.64	BM, DFT (GGA)	Ref.[7]
67.2	266	3.79	BM, DFT	Ref.[8]
66.9	249	4.08	BM, DFT (PBE)	Ref.[9]
65.9	259.3	4.06	BM, DFT(PBEsol)	Ref.[9]
67.4	256.9	3.95	BM, DFT (GGA)	Ref.[2]
64.4	270.8	4.03	BM, DFT (LDA)	Ref.[2]
66.9	242	4	BM, DFT (GGA)	Ref.[10]
65.9	261.1	4.81	BM,DFT (B3LYP)	Ref.[11]
66.9	243.3	4.30	BM, (PBE)	Ref.[11]

[a]The equation of state along the isentrope is calculated assuming Y and γ_0 are 20 GPa and 1, respectively. BM = Birch-Murnaghan EOS, Vinet= Vinet EOS, DFT=density functional theory. LDA, GGA,PBE, PBEsol, and B3LYP designate the names of the exchange-correlation functional used in theoretical calculations.

Supplementary Table 4. Thermodynamic parameters and Taylor-Quinney coefficient of SiC used for the energy and temperature calculations.

γ_0	q	Y (GPa)	V_0 (Å^3)	θ_D (K)	f_{TQ}	E_{Tr} (MJ/kg)
0.5-2	1*	5-20	66.3[b]	1200[b]	1*	3.38[c]

γ_0, V_0, are the ambient Grüneisen parameter, specific volume for B1-SiC. q is the logarithmic volume dependence of γ. Y is the yield strength, θ_D is the Debye temperature of B1-SiC and f_{TQ} is the Taylor-Quinney coefficient.

*Parameters are fixed.

[a]Ref.[12], [b]Ref.[3], [c]Ref.[4].

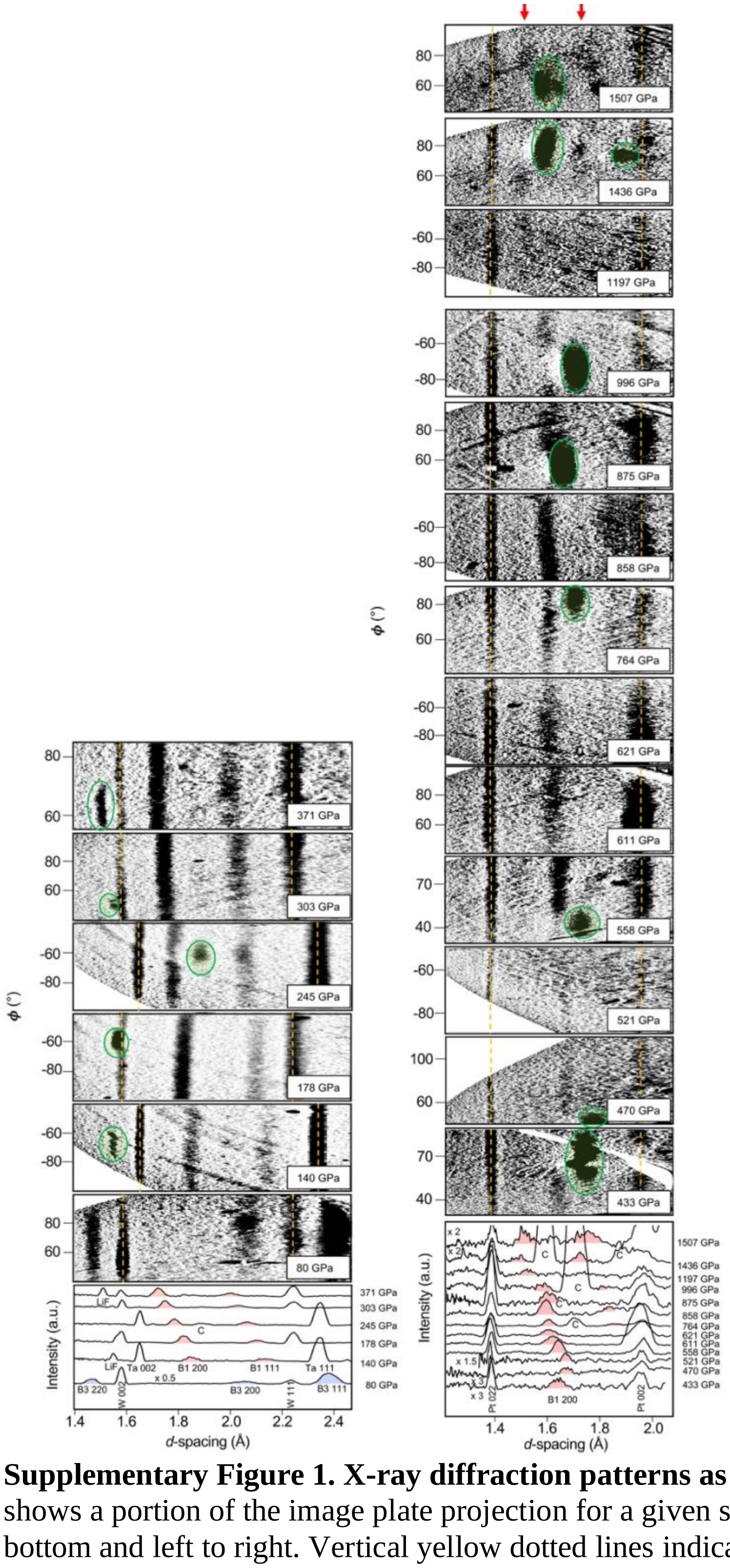

Supplementary Figure 1. X-ray diffraction patterns as a function of stress. Each panel shows a portion of the image plate projection for a given shot, with stress decreasing from top to bottom and left to right. Vertical yellow dotted lines indicate diffraction from pinhole substrate. Green ellipses mark single-crystal diffraction spots from the diamond or LiF. 1-D integrated

diffraction patterns are shown at bottom with B1 and B3 SiC highlighted in red and blue, respectively.

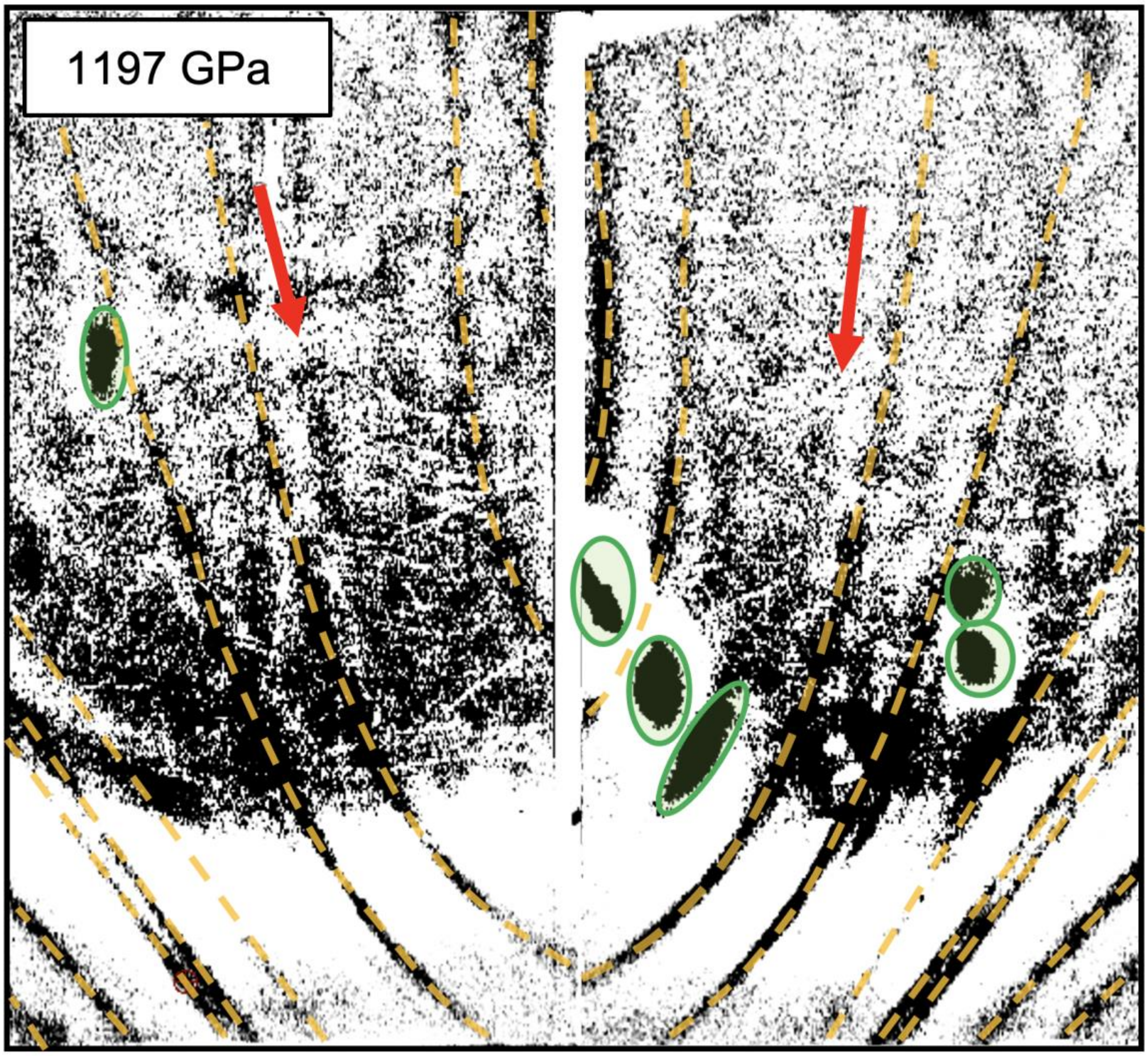

Supplementary Figure 2. Example of image plates from shot s89314 (1197 GPa) after background subtraction. Red arrows indicate diffraction feature from B1 SiC. The green ellipses mark Bragg peaks or Laue spots from the diamond single crystal. The yellow dashed curves correspond to diffraction from the Pt pinhole.

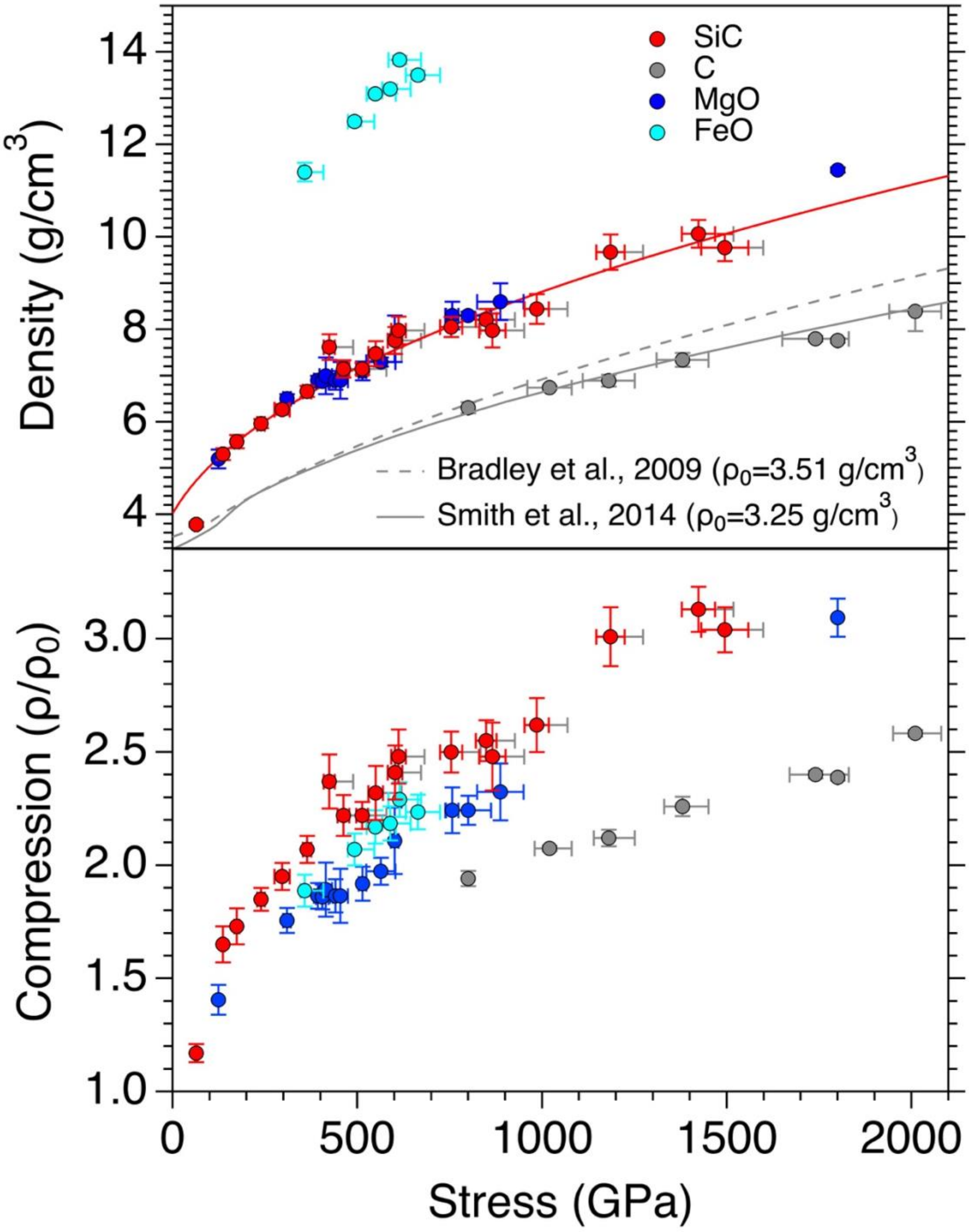

Supplementary Figure 3. Measured densities and compression under ramp loading for candidate planetary mantle constituents at extreme conditions. Gray curve and dashed line indicate two different initial densities of diamond under ramp compression measured at the continuum level[13,14]. Circle symbols show studies reporting *in-situ* X-ray diffraction data[15–17]. Our data for SiC are the highest-compression diffraction data yet reported under ramp compression.

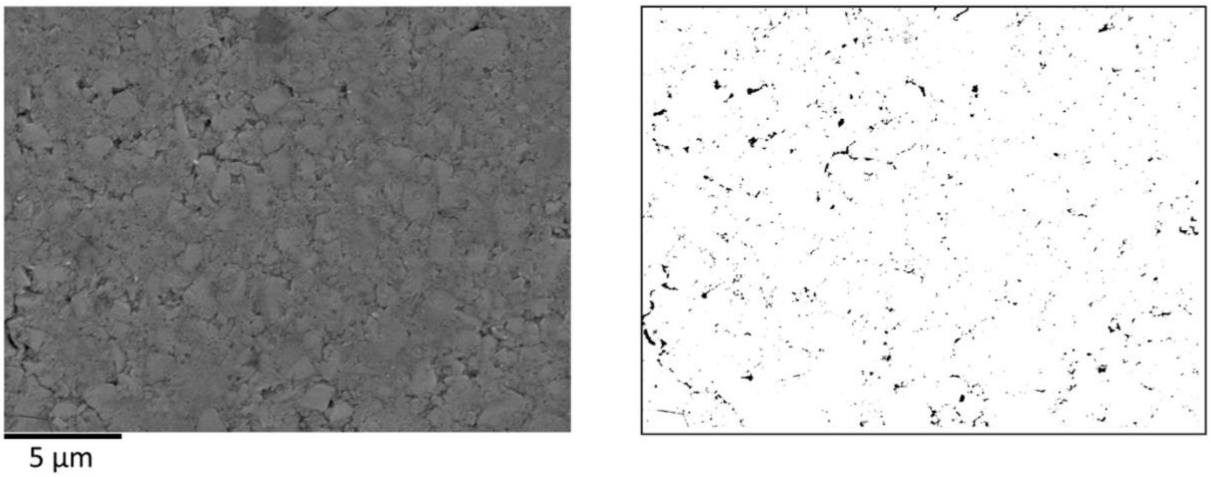

Supplementary Figure 4. Left: Scanning electron microscope image of cold-pressed SiC powder used in ramp-compression experiments. Right: The pore space is identified and shown as dark color using the method in ref.[18]. The estimated porosity is ~1.6%.

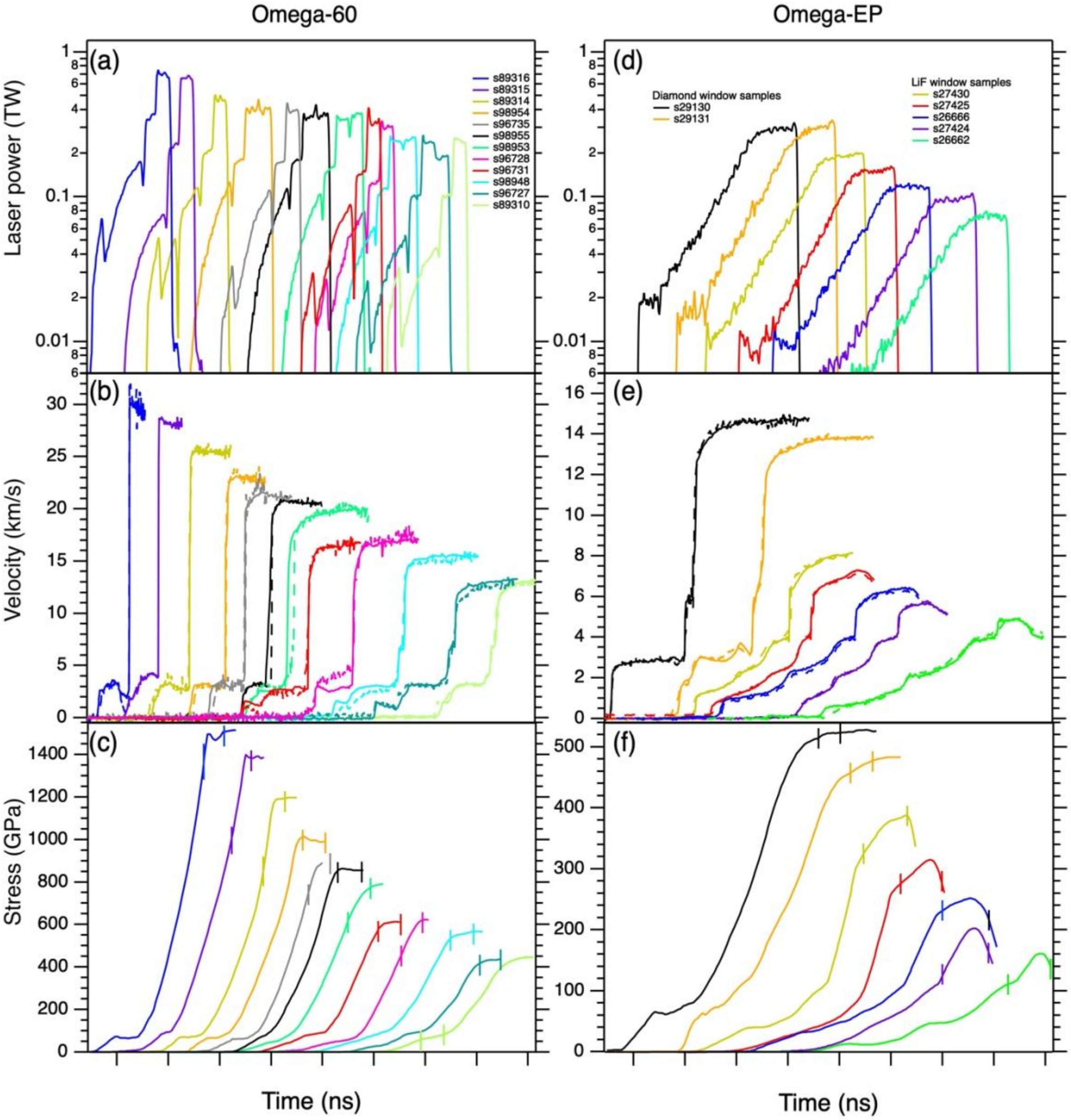

Supplementary Figure 5. Summary of laser power, free-surface velocity and interface velocity, and sample stress. Stress history for SiC ramp-compression experiments at Omega-60 (left) and Omega-EP (right). (**a,d**) Laser pulse shapes (**b,e**) Diamond free-surface velocity and sample-LiF interface velocity traces. (**c,f**) Sample stress/pressure histories as a function of time. Vertical tick marks indicate X-ray probe time. Traces are offset in time for clarity. Tick marks on the x axis correspond to 2 ns.

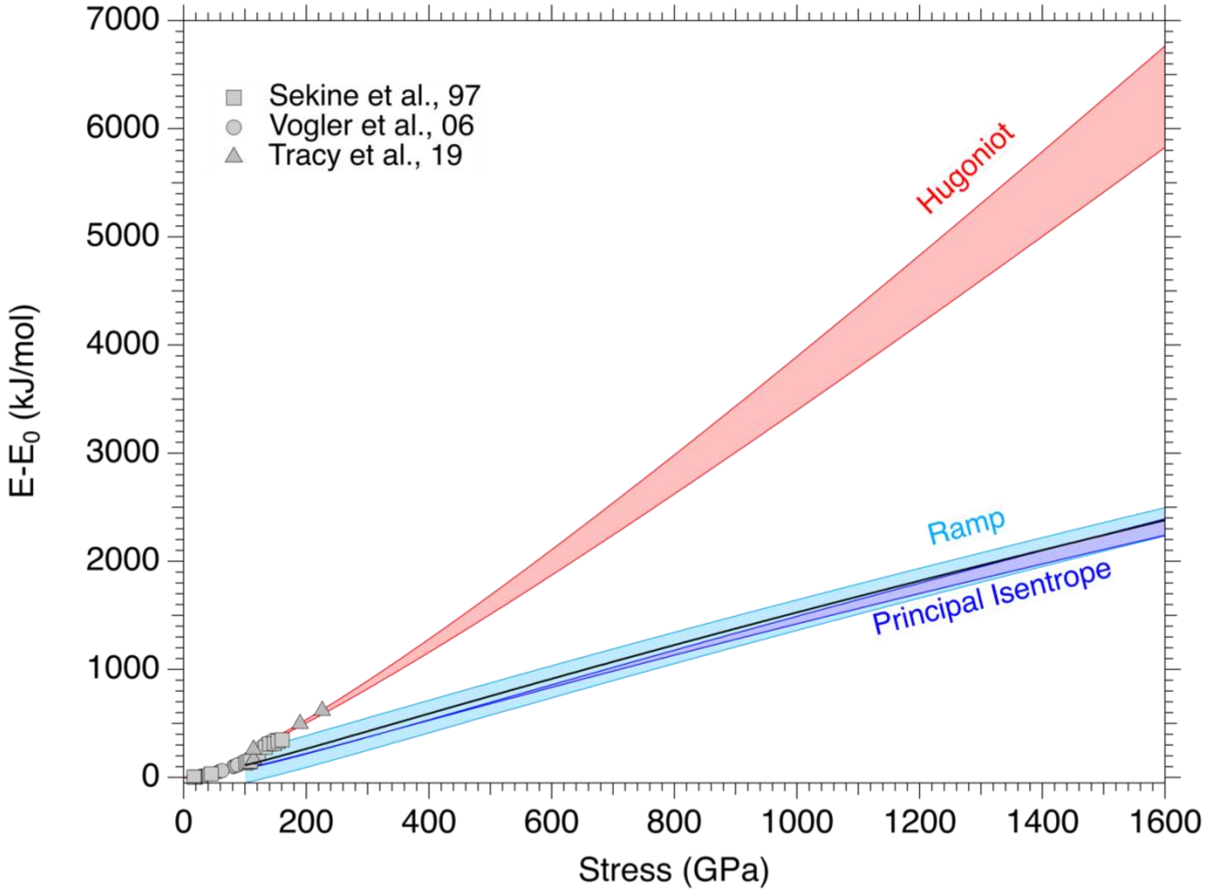

Supplementary Figure 6. Energy along the Hugoniot, ramp and principal isentropic paths as a function of stress. The Hugoniot data[12,19,20] are shown as gray symbols. The red and dark blue bands represent the stress-energy paths along the Hugoniot and principal isentrope, respectively. The black curve and light blue band indicates the stress and energy paths under ramp loading including the sum of energies from the initial shock wave in the sample accounting for porosity, deviatoric stress, the plastic working heating, and B3-to-B1 phase transformation energy. The black curve used for ramp temperature calculation (Supplementary Figure 7) was obtained assuming Y = 20 GPa and γ_0 = 1. The thermodynamic parameters used are listed in Supplementary Table 5. The large uncertainty in the ramp energy is due to the wide range of plausible strength and thermodynamic parameters of B1 SiC at ultrahigh pressures. Better constraints on these quantities from experiment and theory are needed.

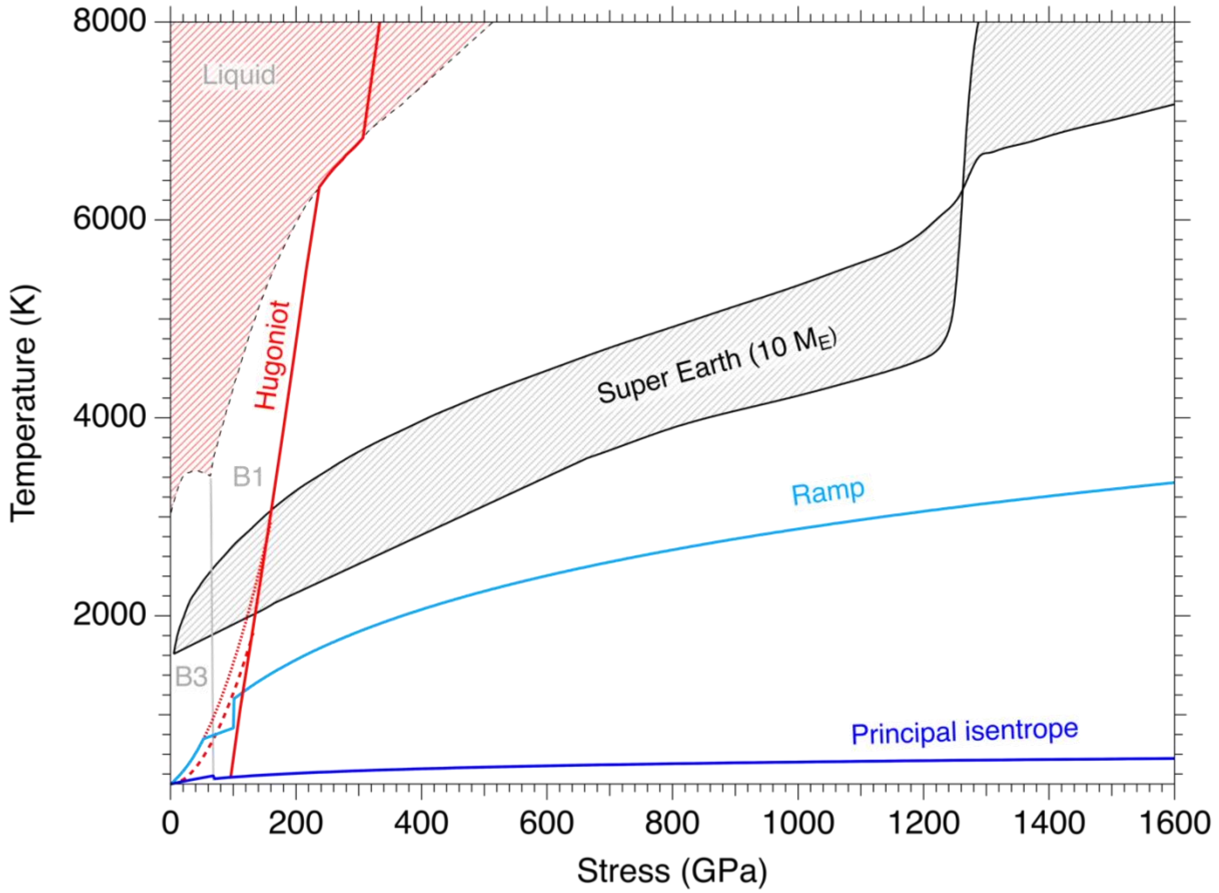

Supplementary Figure 7. SiC phase diagram with phase boundaries[3], Hugoniot, predicted thermodynamic paths and stress-temperature conditions for a ten-Earth-mass rocky planet. The blue curve indicates the stress and temperature paths along the principal isentrope. The light-blue curve and dashed curve show the path taken for an initial shock (~52 GPa) followed by isentropic compression of B3 (52-100 GPa) and the estimated ramp paths of B1 (above 100 GPa) assuming Y and γ_0 are 20 GPa and 1, respectively, corresponding to the stress-energy path shown as the black curve in the Supplementary Figure 6. The dotted and dashed curves represent the stress and temperature along the Hugoniot of B3 with porosity (2%) and full density respectively. Stress-temperature range for a 10-Earth-mass rocky exoplanet are shown as the gray band[21,22].

Supplementary References

1. Coppari, F. *et al.* Optimized X-ray sources for X-ray diffraction measurements at the Omega laser facility. *Rev. Sci. Instrum.* **90**, 125113 (2019).
2. Kidokoro, Y., Umemoto, K., Hirose, K. & Ohishi, Y. Phase transition in SiC from zinc-blende to rock-salt structure and implications for carbon-rich extrasolar planets. *Am. Mineral.* **102**, 2230–2234 (2017).
3. Miozzi, F. *et al.* Equation of state of SiC at extreme conditions: new insight into the interior of carbon-rich exoplanets. *J. Geophys. Res. Planets* **123**, 2295–2309 (2018).
4. Karch, K., Bechstedt, F., Pavone, P. & Strauch, D. Pressure-dependent properties of SiC polytypes. *Phys. Rev. B* **53**, 13400–13413 (1996).
5. Durandurdu, M. Pressure-induced phase transition of SiC. *J. Condens. Matter Phys.* **16**, 4411–4417 (2004).
6. Durandurdu, M. *Ab initio* simulations of the structural phase transformation of 2H-SiC at high pressure. *Phys. Rev. B* **75**, 235204 (2007).
7. Lu, Y.-P., He, D.-W., Zhu, J. & Yang, X.-D. First-principles study of pressure-induced phase transition in silicon carbide. *Physica B: Condens. Matter* **403**, 3543–3546 (2008).
8. Wilson, H. F. & Militzer, B. Interior phase transformations and mass-radius relationships of silicon-carbon planets. *Astrophys. J.* **793**, 34 (2014).
9. Lee, W. H. & Yao, X. H. First principle investigation of phase transition and thermodynamic properties of SiC. *Comput. Mater. Sci.* **106**, 76–82 (2015).
10. Gorai, S., Bhattacharya, C. & Kondayya, G. Pressure induced structural phase transition in SiC. *AIP Conf. Proc.* **1832**, 030010 (2017).
11. Gorai, S. & Bhattacharya, C. Shock induced phase transition in SiC polytypes. *J. Appl. Phys.* **125**, 185903 (2019).
12. Tracy, S. J. *et al. In situ* observation of a phase transition in silicon carbide under shock compression using pulsed X-ray diffraction. *Phys. Rev. B* **99**, 214106 (2019).
13. Smith, R. F. *et al.* Ramp compression of diamond to five terapascals. *Nature* **511**, 330–333 (2014).
14. Bradley, D. K. *et al.* Diamond at 800 GPa. *Phys. Rev. Lett.* **102**, 075503 (2009).
15. Lazicki, A. *et al.* Metastability of diamond ramp-compressed to 2 terapascals. *Nature* **589**, 532–535 (2021).
16. Coppari, F. *et al.* Implications of the iron oxide phase transition on the interiors of rocky exoplanets. *Nat. Geosci.* **14**, 121–126 (2021).
17. Coppari, F. *et al.* Experimental evidence for a phase transition in magnesium oxide at exoplanet pressures. *Nat. Geosci.* **6**, 926–929 (2013).
18. Howes, B., Mehra, A. & Maloof, A. Three-dimensional morphometry of ooids in oolites: a new tool for more accurate and precise paleoenvironmental interpretation. *J. Geophys. Res. Earth Surf.* **126**, e2020JF005601 (2021).
19. Vogler, T. J., Reinhart, W. D., Chhabildas, L. C. & Dandekar, D. P. Hugoniot and strength behavior of silicon carbide. *J. Appl. Phys.* **99**, 023512 (2006).
20. Sekine, T. & Kobayashi, T. Shock compression of 6H polytype SiC to 160 GPa. *Phys. Rev. B* **55**, 8034–8037 (1997).
21. Wagner, F. W., Tosi, N., Sohl, F., Rauer, H. & Spohn, T. Rocky super-Earth interiors: structure and internal dynamics of CoRoT-7b and Kepler-10b. *Astron. Astrophys.* **541**, A103 (2012).

22. Duffy, T. S. & Smith, R. F. Ultra-high pressure dynamic compression of geological materials. *Front. Earth. Sci.* **7**, 23 (2019).
